# 

**Published:** 1922-04

**Authors:** 


### RECENT APPOINTMENTS.

### Public Health.

Dr. F. M. S. Hulke, to be M.O.H. for the Borough of Deal.

Dr. Howell Wood Barnes, B.A., M.B., to be M.O.H. for the Borough of Camberwell.

Miss Beatrice Garvie, L.R.C.P., L.R.C.S., (Edin.), D.P.H., to be assistant to the Medical Officer of Health for Islington.

Dr. Aubrey Middleton Hewat, M.D., M.B., Ch.B., D.P.H., M.O.H. and School Medical Officer at Nuneaton since August, 1919, to be M.O.H. for the Metropolitan Borough of Fulham.

Dr. Arthur Gordon Ord and Dr. James Carlisle Davis, to be respectively First and Second Resident Medical Officer for the Portsmouth Workhouse Infirmary, Workhouse and Children's Home.

### Institutional.

The Rev. E. H. Dunkeley has been appointed chaplain to St. Bartholomew's Hospital.

Miss M. V. Winters has been appointed matron of the Royal Infirmary, Truro. Previously she held the post of tutor sister at the Nottingham.

Mr. James Higson has been appointed chairman, and Mr. Leonard Sumner deputy chairman, of the Salford Local Committee under the Voluntary Hospitals' Commission.

Mr. J. H. Bryan has been appointed secretary of the Bournemouth Saturday and Sunday Fund, in succession to Mr. W. Bennett, who has resigned after ten years' service.

Miss E. M. Lucy has been appointed an assistant almoner with the care of women attending the V.D. clinic at King's College Hospital, where the number of almoners has been reduced.

Mr. J. M. de Vine, for twenty years general superintendent of the Royal National Hospital, Ventnor, has received a presentation from the board on his retirement. Dr. Hutchinson has been appointed medical superintendent.

Dr. John Macarthur, at present medical superintendent of Colney Hatch Mental Hospital, will succeed Dr. Johnston as medical superintendent of Bracebridge Mental Hospital, in May. Dr. Johnston's retirement is due to ill-health.

Mr. Arthur Griffiths, O.B.E., Superintendent of the East Suffolk and Ipswich Hospital, has been appointed honorary secretary of the Local Committee of the County of Suffolk under the Voluntary Hospitals' Commission. The meetings will be held at the Ipswich County Hall.

### THE ROYAL INSTITUTE OF PUBLIC HEALTH.

We have received a copy of the preliminary programme of the Plymouth Conference of the Royal Institute of Public Health (May 31-June 5). Its work is to be divided into four sections. In Section I., "State Medicine and Municipal Hygiene," the topics to be discussed include the unification of local health administration, the prevention of tuberculosis from human and bovine sources, the improvement of the milk supply, medical and social problems of industrial hygiene, how to raise the standard of housing through the exercise by local authorities and sanitary officers of their ordinary powers, the administrative control of diphtheria, and the prevention of venereal disease; Section II., "Naval, Military and Air," will cover the subjects of physical training and recreation from the point of view of public health, the destruction of animal carriers of disease, health education, war wastage through minor ailments, and points of interest respecting preserved food, more particularly that supplied to the Fleet.

In Section III., "Bacteriology and Bio-chemistry," the papers to be read include "Botulism and Canned Fruit," and "Recent Advances in the Bacteriological Examination of Water"; and Section IV., "Women and Public Health," will deal with maternity and infant welfare work, the school child, and the racial and industrial aspects of maternity.

### PARLIAMENTARY QUESTIONS AND ANSWERS.

February 27.—Sir A. Mond, in reply to questions by Sir Alfred Davies, Mr. Hayden and Mr. Grundy with reference to amendments of rules passed by the General Nursing Council, said: "The Chairman of the General Nursing Council consulted me as to these amendments, and I informed him that I was prepared to sanction any rules consistent with the Act which the Council thought necessary to speed up registration and to secure an adequate electorate before the first elections to the Council are required by the Act to be held. The rules already provide for the annual re-election of committees in the second and subsequent Councils, and the extension of this provision to the present Council is in accordance with the usual practice of similar bodies. The Council considers that a reconstitution of existing committees will expedite the formation of the register, and the experience of the last six months leads me to share their view. I strongly deprecate the suggestion that the matrons on the Council represent the interests of the employers, or that they are any less solicitous than other nurses for the welfare of the nursing profession, and I see no reason whatever for thinking that the new rule will be detrimental to the interests of working nurses."

March 1.—Sir A. Mond informed Mr. Hurd that he was considering the question of introducing legislation on the subject of milk; and stated in reply to Mr. Stewart that, as at present advised, he was not prepared to suggest legislation making the notification of venereal disease compulsory.

Sir A. Mond stated in reply to Captain Elliot that during the current financial year grants of £9,500 had been made to the National Council for Combating Venereal Disease.

March 7.—In reply to Sir R. Clough, Sir A Mond stated that returns from approved societies comprising about half the total number of insured persons showed an increase of sickness benefit claims during 1921 of 1·4 in the case of men, and 8·4 in the case of women. He attributed this in part to the influenza epidemic towards the end of the year.

March 13.—Major Tryon informed Captain Bowyer and Mr. Pennefather that the offer to the Hammersmith Guardians of an increased rent of £12,000 a year for the Orthopædic Hospital, Shepherd's Bush, was still open.

March 14.—Sir A. Mond stated in reply to Sir F. Hall that the Insurance Bill shortly to be introduced would not in any way modify the relations of doctors and insurance committees as regards the administration of medical benefit.

Sir A. Mond informed Mr. Mills that no attempt had been made to press Boards of Guardians or other local authorities to adopt the Schick test. He added that the test marked a great advance in our methods of prevention and control of diphtheria.

March 15.—Sir A. Mond, in answer to Colonel Wedgwood and Mr. Ammon, denied that any experiments in connection with the Schick test were being conducted upon children in poor-law schools.

Sir A. Mond informed Colonel Burn that the new Insurance Bill would provide that the cost of medical benefit, at present met out of the Exchequer, should be defrayed until December 31, 1923, from approved society funds, but the Bill would not entail any changes in the control of medical benefit.

March 16.—Sir A. Mond informed Mr. Maclean that information in the possession of approved societies relating to insured persons could not be used for purposes unconnnected with insurance without the insured persons' consent; but that in special cases his Department informed societies that information should be supplied where it was necessary in the interests of justice.

### MINISTRY OF HEALTH ESTIMATES.

February 27.—Supplementary Estimates required by the Ministry of Health for the financial year just ending were voted. The vote itself was for a token sum of £10, the amount actually required (£1,587,000) being met out of the savings (£3,800,000) on administration, housing and insurance, the savings on the latter item being due in part to the reduction of the doctors' remuneration as from January last, and in part to smaller sickness claims. An additional sum of £514,000 was required for tuberculosis. The estimates were the subject of some criticism, especially in connection with the sums required for the cancellation of contracts for bricks to be used on housing schemes.

March 13.—On the vote on account of £9,000,000 for the coming financial year for the Ministry of Health, Mr. Clynes moved a reduction of £100. The hon. member devoted his speech to a criticism of the Government's housing policy, and other members followed on the same lines. Mr. G. Locker-Lampson urged the amalgamation of the Health and Unemployment Insurance machinery and the abolition of the Welsh Board of Health. Sir A. Mond in reply said that it had been decided not to adopt the recommendation of the Geddes Committee on the latter point, and stated that an expert committee was examining the possibilities of amalgamating the two insurance schemes. With regard to housing he pointed out that the housing scheme would cost £10,000,000 a year for many years, the result of over-stimulation at the beginning of the housing programme, which caused prices to be forced up. The cutting down of the Government programme had already caused a substantial fall in prices, and he was hopeful that private enterprise would soon get to work again. The shortage of plasterers was accentuating present difficulties. The debate was ocntinued by Mr. Myers, Lt.-Col. Fremantle, Sir Watson Cheyne (who urged the importance of milk legislation), Lord Robert Cecil and others, and the reduction in the vote was finally negatived by 156 to 52.

### NEW BILLS AND LEGISLATIVE PROPOSALS.

February 28.—The Coroners (Emergency Provisions Continuance) Bill has received the Royal Assent. This Bill extends, until December 31 next, war-time enactments enabling coroners to hold inquests without a jury in certain cases, and reducing the maximum and minimum number of jurymen to 12 and seven respectively. The Home Secretary, in moving the second reading of the Bill, stated that the whole subject was now under consideration with a view to permanent legislation.

March 1.—The House of Commons, by 234 to 86, refused leave to Major Lowther to bring in a Bill for the abolition of capital punishment.

March 15.—Mr. Inskip obtained leave to introduce a Bread Acts Amendment Bill, for the purpose of making it clear that the use of self-raising flour was not unlawful. He stated that the clauses in the Bill had received the assent of the Minister of Health, whose powers under the adulteration of Food and Drugs Acts were preserved. The Bill was read a first time.

The Clinical Thermometer Bill has been passed by the House of Lords and sent to the Commons. The Bill reproduces certain conditions in Dr. Addison's ill-fated Ministry of Health (Miscellaneous Provisions) Bill and forbids the sale of thermometers unless they have been previously tested to the satisfaction of the Board of Trade. It will be recollected that a similar prohibition was enforced during the war under a D.O.R.A. regulation, which is now no longer operative.

February 27.—Mr. Devlin moved an address to the Crown praying that the Government of Ireland (Adaptation of Health Insurance Acts) Order, 1922, might be annulled, After some discussion the House was counted out. The Order came into force in Northern Ireland on March 1, as from which day Health Insurance will be administered by the Northern Ireland Government instead of the Irish Insurance Commissioners, as heretofore. The Commissioners continue to administer the Acts in the Irish Free State.

On March 2, Mr. G. Locker-Lampson moved an Address to the Crown praying that the Order in Council made under the Dentists Act, 1921, dated January 16, approving Regulations of the Dental Board of the United Kingdom, might be annulled. The hon. member criticised the annual fee of £5 required by the Regulations as a condition of remaining on the Register, and contrasted the practice of the General Medical Council, who only require a fee at the first registration. Mr. Acland, Chairman of the Dental Board, defended the regulations, and eventually the House was counted out. A further address for the same purpose was moved a few evenings later by Mr. Seddon, but after a vigorous defence of the Regulations by Sir A. Mond was withdrawn.

### OFFICIAL REPORTS RECEIVED.

Special Report of the County Medical Officer of Health on the Outbreak of Smallpox in the Borough of Middleton in 1920.

Royal Maternity Charity of London, 1921.

Central Council for District Nursing in London: Seventh Annual Report (1921).

League of Nations: Report on the Health Situation in Eastern Europe in January, 1922.

**Hospitals :—**

Royal Edinburgh Hospital, Morningside (Craig House and The West House), 1921. By George M. Robertson, M.D., F.R.C.P.Ed., Physician-Superintendent and Professor of Psychiatry in the University of Edinburgh.

Bradford Royal Infirmary, 1921.

County Mental Hospitals, Stafford, Burntwood and Cheddleton. Annual Reports, 1920, and Statements of Accounts, 1920-21.

Royal Prince Alfred Hospital, Sydney. Year to June 30, 1921.

Passmore Edwards Cottage Hospital, Liskeard, 1921.

Pontypool and District Hospital, 1921.

West Suffolk General Hospital, Bury St. Edmunds, 1921.

Aberdeen Royal Asylum, 1921.

British Hospital for Mothers and Babies, and School for the Higher Training of Midwives, Woolwich, 1921.

Royal Westminster Ophthalmic Hospital, 1921.

### H.M. STATIONERY OFFICE PUBLICATIONS.

[The prices quoted include postage.]

Ministry of Health Circ. No. 262. Form of Accounts for Approved Agencies for the Blind. 4d.

Ministry of Health Circ. No. 273. Audit Stamp Duty. 2d.

London Government. Minutes of Evidence taken before the Royal Commission, Part I. 3s. 9d.

Building Research Board, Special Report No. 4. The Transmission of Heat and Gases through and the Condensation of Moisture on the Surface of Wall Materials. 1s. 1½d.

Merchant Shipping. Standard of Rejection for Colour-Blindness in Seamen. Report of the Board of Trade. 2d.

Ministry of Health Circ. No. 283. Railway Assessments. 2d.

National Health Insurance Joint Committee (Government of Ireland Act, 1920). Report of the Departmental Committee on the application of the National Health Insurance. (Cmd. 1575.) 7½d.

Schedule of Rules framed under Sections 5 (1) and 6 (1) of the Midwives (Ireland) Act, 1918. 7½d.

Friendly Societies Reports of the Chief Registrar for 1920. Part II, Appendix (N).

Section 1.—Durham, Northumberland, Yorkshire. 2s. 7½d.

Section 2.—Cheshire, Cumberland, Lancashire, Westmorland, Isle of Man. 2s. 1½d.

Section 3.—Derbyshire, Leicestershire, Northamptonshire, Nottinghamshire, Rutland, Staffordshire, Warwickshire, Worcestershire. 2s. 7½d.

Section 4.—Bedfordshire, Berkshire, Buckinghamshire, Cambridgeshire, Hertfordshire, Huntingdonshire, Lincolnshire, Norfolk, Oxfordshire, Suffolk. 1s. 7d.

Section 5.—Essex, Kent, London, Middlesex, Surrey. 2s. 7½d.

Section 6.—Cornwall, Devonshire, Dorsetshire, Gloucestershire, Hampshire, Herefordshire, Monmouthshire, Shropshire, Somersetshire, Sussex, Wiltshire, Channel Islands. 1s. 7d.

Section 7.—Wales. 1s. 1d.

Section 8.—Scotland. 1s. 1d.

Medical Research Council. Report for 1920-21. 3s. 8½d.

Medical Research Council. Use of Death-Rates as a Measure of Hygienic Conditions. (Special Report No. 60.) 3s. 2d.

Industrial Fatigue Research Board. Second Annual Report (to September 30, 1921). 1s. 7½d.

National Health Insurance Commission (Ireland). Form 3. Compensation (Lump Sum). ½d. each; 1s. 6d. per 100.

Ministry of Health Provisional Order (H.L.B. 2). 1s. 1½d.

Clinical Thermometers (H.L.) H.L.B. 6. 3d.

Scottish Board of Health. Summary of Returns made to the Board in terms of Sec. 23 of the Housing Town Planning, &c. (Scotland) Act, 1919, for the half-year to September 30, 1921. Cmd. 1584. 4d.

Catalogue of the National Collection of Type Cultures maintained at the Lister Institute of Preventive Medicine. 1s. 7½d.

Cloakrooms, Washing Facilities, Drinking Water, and Sanitary Accommodation in Factories and Workshops. 1s. 1½d.

Residential Institutions in connection with Maternity and Child Welfare in England. 5d.

Lunacy Reform. Report of the Proceedings of the Conference convened by Sir Frederick Willis, K.B.E., C.B., Chairman of the Board of Control, between Commissioners of the Board and Chairmen of Visiting Hospitals, and Medical Superintendents and Chairmen of Managing Committees of Registered Mental Hospitals and certain others, to consider in what directions Lunacy Administration and the treatment of persons suffering from mental diseases may be improved. 2s. 8d.

Medical Uses of Radium. Studies of the Effects of Gamma Rays from a large quantity of Radium. 5s. 3½d.

Circular to Local Authorities (England and Wales) Section 28 Housing, Town Planning, &c. Act, 1919 (Appeals to the Minister of Health). 2d.

Lunacy (Ireland). 70th Annual Report (Part II, with Appendix) of the Inspectors of Lunatics (Ireland) for 1920. Being the Report of the Houses licensed under the Private Lunatic Asylums (Ireland) Act, 1842, and Mental Hospitals. 1s. 7d.

### Statutory Rules and Orders.

No. 1,926. National Health Insurance. Scilly Isles Insurance Committee Regulations, 1921. 2d.

No. 1,928. Housing, England. Local Authorities (Assisted Housing Schemes) Amendment (No. 2). Regulations, December 17, 1921. 2d.

No. 2,031. Master and Servant. Industrial Diseases. Workmen's Compensation (Twister's Cramp) Order, December 31, 1921. 2d.

No. 1,968. National Health Insurance. Collection of Contributions Regulations (Ireland), December 28, 1921. 3d.

No. 1,969. National Health Insurance. Deposit Contributors Regulations (Ireland), December 28, 1921. 2d.

No. 2,089. Housing, Ireland, Housing (Societies) Amendment Regulations. November 30, 1921. 2d.

No. 2,092/S. 96. National Health Insurance. Medical Benefit Amendment Regulations (Scotland), 1921. No. 3. 2d.

No. 1 (1922). Food Control Order dated January 3, 1922, revoking the Food for Mothers and Children (Scotland) Order, 1918. 2d.

Provisional Regns. National Health Insurance Arrears Amendment Regns. December 31, 1921. 2d.

Provisional Regns. National Health Insurance. Calculation of Contributions Regns. December 31, 1921. 2d.

Draft. National Health Insurance. Arrears Amendment Regns. 1922. 2d.

Draft. National Health Insurance. Calculation of Contributions Regns. 1922. 2d.

Provisional Rules and Orders. Government of Ireland (Adaptation of Health Insurance Acts) Order, 1922. 3d.

No. 110. National Health Insurance. Deposit Contributors Regns. 1922. 2d.

Provisional. National Health Insurance. Approved Societies (Amendment Regulations), dated January 31, 1922, made by the N.H.I. Joint Committee, the Minister of Health and the Irish Insurance Commissioners, acting jointly, and Regulations made by the N.H.I. Joint Committee and the Scottish Board of Health, acting jointly. 3d.

Draft. National Health Insurance. Approved Societies (Amendment) Regulations, 1922. Regulations proposed to be made by the N.H.I. Joint Committee, the Minister of Health, the Scottish Board of Health and the Irish Insurance Commissioners. 3d.

